# ‘Worth the test?’ Pragmatism, pill testing and drug policy in Australia

**DOI:** 10.1186/s12954-018-0216-z

**Published:** 2018-04-10

**Authors:** Andrew Groves

**Affiliations:** School of Humanities and Social Sciences, Deakin University, Burwood campus, Melbourne, Victoria 3125 Australia

**Keywords:** Pill testing, Harm minimisation, Pragmatism, Australian drug policy, Party-drug use

## Abstract

**Background:**

Recent deaths of young Australian music festival attendees from ‘party-drug’ overdoses have sparked debate about the effectiveness of drug policies. Australia is widely lauded for its harm minimisation approach to drugs, and yet, over the last 30 years, it can be argued its policies have been fragmented, sometimes inconsistent and contradictory. The present article examines the root of this inconsistency, using it as a foundation to advocate for drug policy reform. In keeping with the goals of the National Drug Strategy to promote policy innovation, there is an opportunity to learn from international studies which have shown promising findings in the reduction of party-drug use and its harms through application of pill testing.

**Method:**

This paper evaluates Australia’s National Drug Strategy and pill testing through a lens of pragmatism, to determine whether there is space for testing practices in contemporary policy. Specifically, the paper analyses current drug policy literature and research studies, examining a range of key drug use indicators, social and political debate and research evidence.

**Results:**

The need for policy reform, attitudinal and cultural shifts and development of stronger cross-sectoral partnerships is highlighted, to ensure a rational and logical approach that genuinely tackles drug policy-making and strategy from a broad public health perspective.

**Conclusions:**

Using a theoretical frame of pragmatism and drawing from national and international research evidence, this paper recommends the integration of pill testing into Australia’s harm minimisation strategy.

## Background

Young people have long been associated with drug consumption, often displaying patterns of use distinct from the general population [1–3]. Like many other countries, the emergence of dance-music culture and ‘raves’ in Australia in the 1970–1980s bolstered the relationship between drugs and youth, creating dynamic settings in which consumption of so-called ‘party-drugs’ such as methamphetamines, ecstasy and other psychoactive substances has become common [4, 5]. For many young people (i.e. 18–29 years old), attendance at dance-parties and music festivals is a rite of passage within a hedonistic lifestyle where identity and social capital are built, pleasure is ‘consumed’ and alcohol and other drugs (AODs) are ubiquitous. However, youth party-drug use is typically viewed by politicians, criminal justice professionals and the community as deviant, linked to risk-taking, transgression and individual corruption [6], manifest in a range of physical, psychological and social harms [1]. Indeed, there have been several deaths of young music festival attendees in Australia [7–9], which have held youth party-drug use at the forefront of political, social and media agendas. However, notwithstanding the tragic loss of young lives, what is concerning is that these fatal overdoses, and several ‘near-misses’, may have been avoided through more pragmatic and amoral drug policy and practice. Pill testing offers an alternative, yet it remains at the fringe of policy debate, shrouded by punitive praxis and government reticence despite support in the community.

### Policy and practical ‘problems’

Similar to recent experiences in the UK [10, 11] and Europe [12], Australian AOD policy is at a significant juncture. At the policy level, the implementation of the seventh iteration of the National Drug Strategy (NDS) demonstrates commitment to consistent, ongoing national drug policy [13] in response to the problem of drugs, both illicit and illicitly used (i.e. pharmaceuticals, alcohol and tobacco), under the philosophy of harm minimisation. The NDS outlines a series of principles addressing this philosophy, which prioritise delivery of evidence-informed responses, collaborative interdisciplinary partnerships and a trifurcated approach targeting demand, supply and harm reduction [13]. With regard to party-drugs, however, the application of this policy is contested. While the NDS claims the ‘balanced adoption of effective demand, supply and harm reduction strategies’ ([13]:1), in practice, the distribution of resources, action and policy reform across its ‘three pillars’ falls short of this claim. As discussed below, there are considerable funding gaps in AOD treatment [14], zero-tolerance remains the bastion of public policy and resources are principally expended on law enforcement [15, 16]. While in practice, it is not an either/or approach to supply, demand and harm reduction, nor are these domains mutually exclusive, clearly a balanced approach has not yet been achieved.

At the practical level, problems exist regarding the capacity of policy to recognise and respond to emerging patterns of problematic use, where novel, unknown drugs have entered markets [17] at a time when regular users have increased consumption of more potent forms, such as ice (crystal methamphetamine) and MDMA (3,4-methylenedioxymethamphetamine) [18, 19]. The current framework does not fully capture these nuances, constrained by hegemonic notions of abstinence. Instead, the goal should be to reduce the harms that occur when people use these unknown or more potent drugs, given the serious risks. Notably, despite law enforcement efforts and legislative changes [20], current harm reduction initiatives have been largely ineffective [21, 22], evident in monitoring data where certain groups of young people appear to resist social controls by continuing to use party-drugs. As noted in previous studies [23–25], this is because many young people see drugs as playing a normative and peripheral role in their lives, revealing an important transition in patterns of use, where party-drugs have become more mainstream, used by a heterogeneous cohort of ordinary young people [25]. This apparent normalisation has occurred alongside a trend where some users are unaware of what they are taking, engaging in ‘opportunistic’ purchases of drugs at clubs or music festivals rather than prior to events from more trusted networks [26].^1^ Although no use is ‘safe’, these ad hoc practices substantively increase the risks as suppliers are more likely to be strangers, who may have a greater propensity to adulterate drugs with cheaper and/or alternative substances [28, 29]. Reports have increased of ecstasy pills containing large amounts of methamphetamine [30] and other toxic substances (e.g. rat poison), with others recorded as very high-purity [18], which could seriously harm users. In combination, the rise in problematic patterns of use, the emergence of novel substances and imbalanced policy highlight the need for targeted and more pragmatic responses to youth drug use.

### Pill testing/drug checking

Pill testing is a harm reduction strategy used internationally, also known as drug checking or adulterant screening [31, 32], which emerged in the early 1990s in the Netherlands [33] where it is now part of official national policy. Similar initiatives have since been implemented in other European nations including Sweden, Switzerland, Austria, Germany, Spain and France, albeit primarily administered and funded privately [12, 34]. Organisations such as *DanceSafe* also operate in the USA focused on harm reduction through peer-education, where a language of pragmatism has been established [34, 35]. Testing involves dance-party and music festival attendees volunteering a sample of their drugs for analysis by scientists, who provide information concerning composition and purity [32]. In Europe, this is typically undertaken in mobile facilities located near or inside venues to allow timely feedback to users (approx. 30 min). Results are then ‘posted’ anonymously on information boards or event websites (often using red/yellow/green colour-coding), so users can review feedback clearly and discreetly. These practices are possible through partnerships between event promoters, healthcare services and local police and a strong harm reduction philosophy [36, 37]. Most importantly, this approach has the capacity to influence consumption behaviour where, in contrast to relying on the strength of broad anti-drug campaigns, testing in situ can alter behaviour at the time of consumption, primarily shaped by peers and social networks [38], including health workers [39, 40]. Testing can also involve offsite analyses prior to events, encouraging planning among users, though it is less common as these services often require users to provide identification, increasing the perceived risks of being identified by police [41].

Pill testing is well supported at the local level in Europe, with self-report data from users, accounts from key stakeholders (including police) and wider community endorsement that it provides ‘safer’ drug settings by warning users about harmful and/or unexpected substances [34, 41]. Although research on its effectiveness is mixed (discussed below), there is practical evidence that pill testing has helped to reduce overdose frequency, improve healthcare services, and increase knowledge of harm reduction principles [34, 41, 42]. Increased publicity for support services, advocacy for public health campaigns and opportunities for monitoring and research are further benefits observed internationally, which have fostered evidence-informed and more effective prevention and treatment [34, 36]. These outcomes have also served to extend discussion beyond notions of individual criminality and morality to encompass social, economic and welfare debates, challenging conventional thinking about concepts like harm, risk and social responsibility by considering social contexts of drug use to understand the relationship that individuals and environments have on drug-related harms [43]. It is important, however, to emphasise that drug use is dangerous and cannot be conceptualised as risk-free, nor is pill testing a ‘silver bullet’, with some well-documented concerns [44]. Instead, this article argues that pill testing needs to be viewed through a lens of pragmatism, where for certain users in certain settings, it is about providing young people with information about drugs and their use so they can make more informed choices to limit the associated harms, as well as making important practical changes to the settings in which drugs are used.

As discussed herein, such thinking appears confronting within the Australian drug policy landscape, where current discourse is dominated by dogma, moral conflict and criminal justice debate. Yet, this has not always been the case, as Australian drug policy has a fragmented history [45–47], shaped by the changing vagaries of various political, social and moral forces. The aim therefore is to determine whether pill testing ‘fits’ within this larger narrative and lay the foundation for more cogent drug policy, providing a valuable national framework that may be applicable to other international policy settings. Through this lens, the article examines Australia’s drug policy framework, evaluating a range of key indicators, current social and political debates, and contemporary research evidence. Together with discussion of previous examples of rational policy-making, this data will be used to offer support and provide a roadmap for implementation of pill testing as a more pragmatic strategy and to contribute to discussion of harm minimisation.

## Methods: The National Drug Strategy: fragmentation, contradiction and pragmatism?

The question of how pill testing would fit within the NDS is thought-provoking because arguably, it could already. The NDS outlines Australia’s response to alcohol, tobacco and other (illicit) drugs and provides a national framework for coordinated action to limit their use and associated harms [1]. The strategy has been committed to this approach since its inception in 1985, established then as the National Campaign Against Drug Abuse (NCADA). As noted in the introduction, the overarching focus and language of the NDS has been the improvement of public health and minimisation of harms associated with drug use [1, 19]. This was a substantive ideological shift away from traditional conceptualisations of drug use and drug users, which prior to the 1980s were often viewed in terms of disease metaphors (i.e. as ‘sick’) or as the behaviour of a deviant underclass [48]. In this way, harm minimisation was a pragmatic response that sought to shift debate (and policy-making) away from moral judgements about drug use [49]. It was a pivotal moment in Australian policy, signifying the recognition that because drugs have become a persistent feature of contemporary society, an innovative approach was needed to reduce drug-related harms, rather than simply criminalise users. Demonstrating this, one of the priorities of the 2017–2026 strategy is to prevent and reduce adverse health, social and economic consequences associated with AOD use, by

‘providing opportunities for intervention amongst *high prevalence or high risk groups and locations*, including the implementation of *settings-based approaches to modify risk behaviours*…systems to facilitate greater diversion into health interventions from the criminal justice system, particularly for…*young people* and other at-risk populations who may be experiencing disproportionate harm…[and a]…focus on evidence-based strategies shown to reduce alcohol and other drug hospital presentations, reduce the spread of blood-borne virus, decrease road trauma…and *decrease overdose risk*, with translation of this evidence to address new and emerging issues’ ([13]:23, emphasis added).

Many of these goals are consistent with the rationale for pill testing. So, while their achievement using this approach would not be without difficulty and would require cooperation between law enforcement, health and community sectors, such interdisciplinary partnerships, are already claimed as a success of the previous iteration of the NDS [13], as well as initiatives in other countries [41]. Why then, is there reticence among policy-makers to integrate pill testing into current Australian policy and practice?

This conservativism is symptomatic of a larger malaise in Australian crime control, where in recent decades, drug policy can be described as fragmented and contradictory [45–47]. Similar to the penal policies in the UK and USA in the late 20th century, Australian policy has been increasingly volatile and incoherent, fluctuating—often abruptly—between what Garland ([46]:450–9) characterises as adaptive strategies, focused on prevention and partnerships, and strategies of denial, which stress enhanced state control and expressive punishment. These swings are the result of the normalisation of high crime rates and the state’s acknowledgment of their inability to remedy this problem, creating a predicament for governments [46, 47]. As explored by O’Malley ([45]:181), this predicament is shaped by a ‘recurring ambivalence’ where governments seek to divest themselves of the chief responsibility for the delivery of crime control but recognise the political consequences of doing so. This is an enduring dilemma that helps to explain the fragmented and contradictory nature of recent policy. Indeed, the essence of Garland’s argument remains as valid as it did more than 20 years ago as contemporary governments continue to struggle with various ‘crime problems’ (e.g. illicit drugs), in a politicised policy and social landscape where the state is ‘confronted by its own limitations’ ([46]:462), manifest in the perceived failure of criminal justice agencies and the state generally to control crime.

Garland’s framework resonates further with Australian drug policy where, in an attempt to decentralise control but without undermining law and order agenda, politicians and other key actors have altered the discourse of drug policy and criminal justice debate by focusing on the effects of drug use rather than its causes [45, 47]. For example, a recent national campaign features content illustrating the effects of illicit drugs on victims, describes the costs for the community and draws on community fears of crime [50]. This discursive shift has several implications for how drug use is understood and regulated by the state. Firstly, this approach shows that while adaptive strategies are possible, such as prevention initiatives and partnerships between police and healthcare providers, for certain groups of offenders (i.e. drug users), they are often ‘politically difficult and institutionally radical’, susceptible to moral opposition, failures of political will and conflicts of partisan politics ([47]:348, [51]). This results in policy that is inconsistent and vulnerable to changing political and public interests.

Secondly, by focusing on the effects on victims and the community and exposing debate to the vagaries of politics and the media, this approach positions the needs of society against those of the individual. Bull and colleagues [52] argue that this sets a path for policy where the objectives of support services and police conflict, and where harm minimisation goals become linked to more intensive, zero-tolerance policy, reinvigorating the debate about drugs as a problem of moral values. Placing the harms to society in opposition to, or above the harms to users, has the added consequence of the exclusion or ‘othering’ of drug users, in effect curtailing notions of social citizenship [46]. This has a much broader bearing on our understanding of crime and its control, not merely drug policy, as it creates a tension between two contradictory criminologies: of ‘the self’ (where the offender is rational and unremarkable) and of ‘the other’ (who is the dangerous outcast) [45, 47]. This duality produces two distinct but related possible responses by the state: denial of responsibility for the problem and the increased use of punishment as evidence of ‘doing something’. This article shows that the Australian Government appears to have employed both responses in relation to the problem of party-drugs, with consequences for pill testing initiatives.

The challenges posed by pill testing reflect broader difficulties faced by policy-makers in balancing the goals and perceptions of public health and criminal justice responses to drugs. These stem partly from the duality of Garland’s criminologies, where despite conceptualisation of the ordinary, rational offender, for certain crimes such as drug use the field of crime control is largely shaped by a ‘collective experience of…insecurity’ regarding the ‘other’ ([47]:347). Policy then, is often emotive, dominated by campaigns displaying graphic imagery of abuse, dependence and addiction [50, 53]. Similarly, calls for reform are often used by politicians and the media as opportunities to (re)activate moral debates. A legacy of the 20th century is that the drug problem is seen as a ‘war’ to be won [24], so in-line with increased anxiety about crime generally, drug policy has become a political tool through which zero-tolerance principles have flourished. For instance, research evaluations of recent advertising campaigns reveal most participants reported abstinence as the primary message conveyed [51]. The government has, in effect, displaced responsibility to users and their families to reduce drug-harms by avoiding ‘bad choices’ or ‘just saying no’. This has followed a period of largely conservative policy-making overwhelmed by supply reduction strategies, with far greater funding (65%) directed to law enforcement (e.g. roadside testing, diversion), compared with harm reduction initiatives (2.2%) [15]. In relation to party-drugs, this has meant that while some valuable programs have been implemented, including the provision of ‘chill-out’ spaces and medical services at events [54], overall, programs *for* users have been limited. Moreover, while there is merit in an economic argument, the power of this data is its demonstration of an inability to control crime, the exclusion of users and a punitive approach that, despite evidence of its ineffectiveness [55, 56], is ‘too inscribed and too politically potent to be easily dismantled by rational critique’ ([46]:450). However, historically, pragmatic reform in the area of Australian drug policy is possible.

### Pragmatism: looking back to move forward?

As noted in the introduction, Australia’s drug policy domain is contested. In contrast to punitive criminal justice strategies, there have been initiatives successfully trialled and implemented nationally that follow principles of harm minimisation and public health. These examples are central to the arguments presented herein, because they demonstrate effective praxis, as well as give shape to the theoretical lens through which this paper is viewed. Specifically, they address what Rhodes terms the ‘risk environment’ [43], that is, the need for emphasis on public health to drive discourse and action away from exclusively targeting theories of individual pathology, toward recognition of the social and environmental influences on behaviour and how problematic activities such as drug use might be better managed through more pragmatic means and cooperation. Drawn from research on HIV infection, Rhodes’ framework [43] is particularly instructive because it can be used to better understand both the epidemiology of drug use, as well as how policy-makers, practitioners and the community might work together to reduce the associated harms. It highlights the need to share responsibility for tackling drug use across the community, given that drug-related harm intersects with criminal justice issues, health, vulnerability and various social problems—complex challenges faced by young people that require interdisciplinary and comprehensive responses. For example, while not without its own criticisms, the introduction of the Illicit Drug Diversion Initiative (IDDI) in 1999 officially signalled the utility of an operational relationship between police, health and support agencies [57]. The IDDI was created to reconcile tensions between these sectors, establish a more positive relationship and develop best-practice in responding to drug use. Among a range of rehabilitation and support programs, the IDDI also fostered development of several harm reduction-oriented policing strategies for local law enforcement, including Arrest Referral Schemes, where police refer minor drug offenders to assessment and education services, in lieu of criminal conviction, which research indicates is beneficial for police and leads to subsequent harm reductions (e.g. fewer days in incarceration) and increased support-seeking behaviour among drug users [52, 58, 59].

Another positive collaboration was marked by the introduction of Needle Syringe Exchange Programs (NSEPs) and the Medically Supervised Injecting Centre (MSIC) in Sydney, the largest capital city in Australia, located in New South Wales (NSW). The NSEPs were first trialled in 1986 [60], with the MSIC established in 2001 [52]. While, historically, there was conflict between police and health workers linked to these initiatives, legislative reforms and changes to NSW police operating procedures encouraged police to ‘exercise discretion; work collaboratively and develop a positive relationship with local NSEPs; and promote the legal operation and positive outcomes of NSEPs to the wider community’ ([52]:311). These changes complemented policy reform within NSW police, where overdose policies were amended to consider community interest and avoid pursuit of minor possession charges in non-fatal overdoses, reforms subsequently adopted by all other states and territories [52]. This has contributed to arguably more effective responses to drug use (see p.12). However, these strategies are not without fault, nor does reform occur in a vacuum, often affected by economic, social and welfare policies and community attitudes within a wider political context. Consequently, making assumptions about the value of pill testing based solely on the introduction of the NSEP and MSIC is inappropriate. While indicative of more pragmatic responses to drug use (e.g. heroin), there were specific conditions that led to their introduction, which are temporally distal from the current context and argument presented. Primarily, the motivation for these initiatives came from general concerns regarding public health and the threat posed by HIV, related to the lack of access to safe injecting equipment and/or spaces and harms associated with needle-sharing [61]. These policies were not necessarily about supporting drug users, but avoiding an HIV epidemic. It is crucial then to acknowledge that similar momentum has not developed for pill testing, where drug use remains an ‘us and them’ problem and users are socially excluded.

Nonetheless, these are examples of pragmatic responses that sought to reduce drug-related harms, as well as foster cross-sectoral partnerships. Moreover, there is evidence some of these initiatives and reforms occurred during the ‘Howard era’, whose term of Liberal-National coalition (centre-right liberal conservative) government spanning more than 10 years (1996–2007) is usually associated with zero tolerance [62]. Alex Wodak, Director of the Alcohol and Drug Service at St Vincent’s Hospital in Sydney, argues the ‘tough on drugs’ narrative and opposition to harm reduction that came to be associated with the Howard Government did not unilaterally translate into practice [63]. While Commonwealth funding was increased for abstinence-oriented treatment and support services [64], the Howard Government contemporaneously delivered—albeit discreetly—enhanced funding for NSEPs [63]. The lessons learned from the NSEPs are discussed further below, but it is clear that, ideologically, much more can be garnered from this and other examples. The message is that, although challenging, it is possible to pragmatically respond to drug use within a heavily politicised policy environment, by better understanding the nature of the problem and the responsibility to address it.

## Results: Key indicators of the need for a more pragmatic approach

Since the emergence of dance-music culture in Australia, a variety of drugs including ecstasy and methamphetamines have been associated with this scene, used by young people to enhance their experiences [65]. The most recent National Drug Strategy Household Survey (NDSHS) report in 2016 revealed 11.2% of Australians aged 14 years and over have ever tried ecstasy with 2.2% reporting use in the last 12 months [19]. Data are similar for use of methamphetamines with 6.3% reporting lifetime use and 1.4% revealing recent use [19]. Although these figures are lower than other western nations [44, 66], and demonstrate stable or declining rates of use, they reveal that more than 2.2 million Australians have used ecstasy, and more than 1.3 million have used methamphetamines in their lifetime. However, it is not the numeric value but the location and nature of use and associated harms that are of most concern. Firstly, although not representative, a sample drawn from the Ecstasy and Related Drugs Reporting System (EDRS) identified that up to 70% of this use occurs within clubs, dance-parties and music festivals [26]. This is supported by the representative NDSHS data, confirming them as important sites of analysis [19].

Secondly, there appear to be significant shifts in the forms of drug use in the dance-party scene, particularly among youth. This follows national trends, where those aged 20–29 are the most likely to have consumed illicit drugs generally, with more than a quarter (28%) reporting use in the previous 12 months [19]. Internationally, the prevalence of ecstasy and methamphetamine use among youth attending dance-parties is greater than general population rates [37, 42], which also describes the Australian experience [3]. Indeed, while overall rates of use of both substances reported in 2013 and again in 2016 represent a decline from peaks in 2007, these results mask the level of drug use among specific youth subgroups which has remained stable or increased. Sindicich and Burns [26] report that although recent users of ecstasy largely reported consistent use, typically two or three times a month, a quarter of the sample reported an increase to weekly use. During this period, similar patterns were identified among current methamphetamine users, with the use of the more potent ‘ice’ more than doubling, and a comparable increase observed in the proportion of users who consumed daily/weekly [65]. Although ecstasy use has not reached the levels observed in 2007, methamphetamine use has surpassed these benchmarks [19]. Again, the value of these findings is less in the absolute numbers and more about the behavioural patterns they suggest: chiefly, increased use of more potent substances, concentrated among a novel youth subgroup.

Equally important is the capacity of monitoring systems to respond to changes in drug markets, in order to track and respond to new groups of users. The primary form of monitoring in Australia is the EDRS, which compares interviews with regular ecstasy and other drug users and key professionals, with several key indicators to map trends in drug use, price, purity and availability. In 2015, the EDRS revealed that ecstasy and methamphetamines were readily available and primarily of moderate quality/purity [26]. For ecstasy, although a third of users reported purity as moderate (35%), with a further 20% reporting high-purity pills, more than a quarter perceived levels to be fluctuating (29%). For methamphetamines, the data followed national trends with a shift toward ice, which was far more accessible (97% reported either ‘easy’ or ‘very easy’) and where purity was rated as either moderate (34%) or high (46%), although this form also experienced the greatest perceived fluctuation (15%) [26]. These figures describe accessible drugs that vary markedly in quality/purity, which is problematic as even moderate variations exacerbate already significant risks. Caution must be taken when interpreting these figures though, as they relate to relatively new and capricious drug use settings (e.g. music festivals). The EDRS also relies on data from sentinel groups of regular users (approx. 800 in 2016), as well as professionals (e.g. GPs, police, treatment providers) who interact with them, to determine consumption patterns [26]. Previous research [24, 25] has revealed that party-drug users, however, are a heterogeneous group of consumers, many of whom are educated, socially and economically stable and who rarely come into contact with criminal justice, treatment or support services. Many do not consider themselves more than ‘occasional’ users [25], so are not captured by existing data collections. In addition, although cross-sectional surveys are effective in evaluating users’ perceptions of consumption habits and online marketplace analysis (e.g. the recently shutdown ‘Silk Road’) [67] has emerged as a contemporary method to track drug sales, because drug samples are not scientifically tested, these perceptions and sales cannot be linked with what is actually consumed [68].

Wastewater analysis is another nascent form of monitoring used in the last decade in Australia [69, 70] which provides data about the level and type of drug use through testing of excreted drug residues in sewage/wastewater. This process is similarly limited in its scope to fully examine and minimise the harms associated with party-drug use. To date, these tests have focused primarily on defined geographical areas and broad population analyses (e.g. large catchment areas in capital cities and rural areas [69]), which prevents the linking of compositional data to what young people think they are taking, and sensitivity to changes in consumption trends of particular groups. Although wastewater analysis has been undertaken at Australian music festivals [71], again, only small-scale population data can be collected as this method is unable to record finer demographic detail. For example, data on gender, age and ethnicity of users, differences in route of administration, the number of users (i.e. occasional use by many or heavy use by a few) and the different forms of drug used (e.g. ice versus speed) cannot be distinguished using wastewater analysis [72]. This method is further constrained by lag-times in data collection and analysis, incomplete databases and its retrospective approach, occurring once drugs have been taken, making it less responsive to market changes and less preventative in terms of the harms experienced and individuals’ decisions to use drugs [67].

Another concern relates to the threats posed by new psychoactive substances (NPS), which have emerged in Australia [30, 68] following rapid rises in Europe [12, 32, 73] and popularity at dance-parties and music festivals. These substances, also known as analogues or synthetics, are designed to mimic established drugs [17] and often comprise new, untested chemicals used by drug manufacturers to replace others either in short supply or banned through changes to possession, production and importation laws. This means their contents and effects are unpredictable, placing users and the community at further risk of harm due to an ever-increasing number of ‘unknowns’. This risk is demonstrated in recent findings from the USA and Canada, where several studies identified the introduction of fentanyl in the illicit drug market [74, 75]. Specifically, evidence suggests a wide range of pills (e.g. MDMA) and other drugs (e.g. methamphetamine, cocaine) have been laced with fentanyl, highlighting the potential danger of relying solely on existing practices and technologies, as often local laboratories or other facilities (e.g. hospitals, police) do not have capacity for fentanyl testing or detection of new analogues [74]. While drug use cannot be conceptualised as ‘safe’, greater knowledge of these substances arguably improves policy and treatment options. In recognition of this, questions regarding NPS were first incorporated into the NDSHS in 2013, where approximately 80,000 (0.4%) of the population indicated lifetime use, primarily 20–29 year olds [67]. This population has increased steadily since [19], although levels of use are likely underreported as these substances are characterised by psychoactive properties that imitate existing drugs. Users may therefore be unaware of what they are taking, confounding both monitoring and treatment efforts. Although no deaths linked to fentanyl have been confirmed in Australia, the presentation of 10 drug-affected youth in one night at Royal Perth Hospital in 2013 [30] demonstrates the devastating consequences of new ‘batches’ of unknown substances. Pill testing then may serve as an additional mechanism through which to maintain pace with shifts in drug use trends and contribute to more effective prevention and treatment. Certainly, pill testing cannot be a stand-alone tool; rather, best practice would be its integration into the current NDS to provide both general data on consumption trends and market fluctuations and specific information to users to reduce drug-related harms.

## Discussion: Research evidence: ‘What works?’

Like most debates about policy reform, a key question in the rationale for pill testing is whether it ‘works’. The literature is complicated and, to date, no studies have fully tested in a controlled way, whether pill testing reduces harms. Most evaluations concern attitudinal change (e.g. what people *would do* [20]), legal issues and the integrity of various analytic procedures, with others describing program features or contextually relevant praxis [76], so although not within the scope of this paper, a large, multi-site systematic review of testing practices is needed. Nevertheless, part of the paradox of pill testing comes from expectations of drug policy and practice generally, where effectiveness is often measured in language of abstinence. As a robust body of literature has shown [48, 77], however, abstinence is a goal that displays ignorance of reality. A much broader definition is needed, which demarcates effectiveness more pragmatically, as any strategy shown to improve public health or reduce the prevalence or severity of drug-related harms. For example, connecting users with support services, increasing education and awareness, monitoring market changes and encouraging avoidance of dependence are strategies shown to be effective in Europe [41, 77]. Despite this, like in the UK [10, 77], Australian policy-makers have appeared to take limited account of these findings. Only recently has meaningful debate begun on some of these issues in an unprecedented drug summit, convened in 2016 by the Australian Parliamentary Group on Drug Policy and Law Reform (APGDPDR). It is too early to gauge the full impact of the summit, other than its symbolic value in bringing together key stakeholders, and their collective agreement that the current approach is not working [78]. It is logical then, to seek further guidance on drug policy reform.

In many ways, Australia’s experience mirrors recent trends in the Netherlands [41], Portugal [79], and Switzerland [37], particularly in terms of rates of ecstasy and methamphetamine use and the emergence of NPS. Over the last 20 years, the political landscapes in these countries have similarly been characterised by growing concerns over the social exclusion and marginalisation of drug users, sparking substantive policy reforms. Although policy transfer is not ‘one-size-fits-all’, influenced by community attitudes, individual rights, broader political structures, and the different ways (drug) problems are experienced [77], much can be learned from these examples. In Portugal, for instance, pill testing was implemented alongside comprehensive changes to policy, discourse and philosophy about their drug problem. Personal possession of all drugs was decriminalised in 2001, following radical shifts in social thinking (akin to Rhodes’ approach [43])––that conceptualised drugs as a public health concern, leading to increased resourcing of prevention, treatment and social reintegration programs [80]. Although attitudes to drugs are more liberal in Europe [41], suggesting caution in any comparative analyses, the literature indicates that, in particular settings, pill testing can reduce the prevalence of harms for users, influence youth decision-making and positively impact drug markets. In terms of the latter, pill testing has been shown to affect the manufacture and distribution of pills [41, 81]. By accurately identifying drug content and purity/potency, the Netherlands’ Drug Information and Monitoring System (DIMS), for example, has informed national warning campaigns, which has pushed dangerous, low-quality substances from the market [41, 81]. Another benefit has been, over time, the composition of tested pills has begun to more closely correspond with expectations [32, 76], increasing overall drug-quality, while alleviating some of the strain on under-funded healthcare and support agencies by reducing the prevalence of overdoses and hospital admissions [15].

Most notably, pill testing has been shown to positively affect users’ behaviour, contradicting claims often used as the rationale for criminalisation that ‘soft’ options encourage increased uptake and use, particularly among youth [68, 82, 83]. Evaluation of the *chEckiT* project in Austria reported approximately half of users whose drugs were tested indicated that information about quality/purity would influence their decision to take them [36]. If presented with a negative result, two thirds reported they would not consume their drugs and would also warn friends against consumption [36, 76]. This corresponds with research from the Netherlands [37], which revealed no increases in the use of most party-drugs (or poly-drug use) because of pill testing and provision of drug information. This also supports evaluations of the reforms in Portugal, where pill testing, as part of a wider public health approach, in fact reduced problematic use, related harms and burden on the justice and healthcare systems [79, 80]. Similarly, when users access testing sites (e.g. at festivals), it allows health and support workers to establish contact with this hard-to-reach population and provide advice about the support available [34]. This is crucial as it is often the first interaction these young people have with any type of support service [31, 37], given they represent a diverse and well-balanced cohort, who are less likely to come into contact with the criminal justice or healthcare systems. Furthermore, party-drug users appear to be highly receptive to harm reduction and prevention measures and/or messages when they are delivered face-to-face and by more trusted sources [42], even among dependent and poly-drug users [37]. As found by several studies, the contact users have with support workers, combined with factual information concerning individual drug purchases and other market information, provide a strong foundation for subsequent health-conscious behaviour [41, 84]. Because young drug users often dismiss government messages as untrustworthy, they are also better persuaded by well-informed peers or professionals [40, 41]. This strategy has long-term benefits, shown to increase users’ motivation for subsequent participation in follow-up counselling sessions [32, 37], providing impetus for support of peer-education and peer-led interventions.

A final feature of pill testing is that it enables monitoring of drug-forms, patterns of consumption and the characteristics of users [37]. The party-drug scene is typified by the use of a large range of substances, the composition of which is expectedly variable and inconsistent. Widespread testing within this setting enables collection of long-term trend data about what users are actually taking, useful for identification of current markets and drug-taking methods [32, 42]. This would in turn build academic research capacity, improve prevention planning and enhance knowledge and research methodology, through directly linking users’ perceptions with their consumption rather than relying on self-report or broad population studies. This may also influence existing supply and demand reduction efforts where, for example, many users report reliance on online networks and/or websites that provide more comprehensive information on drug purity, availability and effects than is available through official sources [82]. The dissemination of more accurate drug information from pill testing, through these online channels (e.g. social media, online forums), could identify and force out of the market websites or dealers found to be sharing inappropriate and/or incorrect information, which is likely impact supply routes, helping police to direct their resources. Beyond this, compared with retrospective analyses (e.g. wastewater analysis), in situ pill testing has the capacity to act as an early warning system to identify the emergence of new drugs more quickly, which is critical given the recent surge in NPS [73, 85]. Overall, these factors allow policy-makers and support services to be more responsive to dynamic market shifts and build knowledge for the development of targeted prevention initiatives. In Australia, however, unquestionably drug policy debate is over-shadowed by philosophical and moral conflict, so for pill testing to be possible requires broader acceptance and a clear direction for its implementation.

## Support in the Australian context

A number of policy models set out a way forward for the introduction of pill testing, which has, in fact, already been trialled in Australia, albeit briefly [86, 87]. In the ‘Enchanted Forest’ raves in South Australia from 2000 to 2001, a group of physicians with the backing of the Australian Medical Association (AMA), several harm reduction non-government organisations (NGOs) and the ‘understanding of local authorities’ examined ravers’ pills in an attempt to reduce consumption [88, 55]. Indicative of the contentious and fragile nature of drug policy though, these trials were terminated after only a short period by the Howard Government [86, 89]. Despite limited opportunity, the research was able to identify large variations in pill composition, emergence of new substances and discrepancies in police testing procedures [88], providing a platform for more comprehensive follow-up, as well as indication of local-level support from experts and health practitioners.

A wealth of empirical data also reveals considerable community support for pill testing, challenging punitive criminal justice responses to drug use. Several studies [76, 90] and the 2013 NDSHS report [65] suggest many Australians see little value in punitive sanctions (e.g. imprisonment, increased fines) for drug use. Instead, referral of users to treatment or education programs appears the preferred response (approx. 45%), with only drug manufacture and distribution perceived to warrant harsh penalties. Drawing from a large (*n* > 2300) internet survey of young Australians, Lancaster and colleagues [76] report the majority back the implementation of pill testing (82.5%), as well as other harm reduction initiatives (NSEPs 76%, ‘chill-out zones’ 65.6%). An even greater level of support was reported in a survey conducted at a major Australian music festival in 2016, where most participants (86.5%) believed testing services could help to reduce harm for users [3]. These findings describe a cohort that values information and seeks to engage in safer practices, regardless of whether they use drugs. Notably, many youth also appear to translate this drug knowledge into behavioural change, with an Australian study finding more than three-quarters of regular ecstasy users would not take an ‘unknown pill’ [91]. A similar result was identified in a more recent sample of users at Australian dance-parties or music festivals [29], where 90% reported seeking information about drug contents in the last 12 months. Most of these respondents (60%) had encountered unexpected substances or problems with drug purity during this period, which motivated them to alter their behaviour with more than half warning friends (51%), many deciding not to consume their drugs (39%) and more than a quarter reducing the amount they consumed (28%) [29]. Most respondents also reported they would use a form of self-testing (94%), onsite event testing (94%) or a fixed-site (i.e. ‘drop-in’) service, and valued services that provided comprehensive, individual feedback rather than only when dangerous results were found. This reinforces previous claims that young people can be persuaded to make rational decisions and are willing to use testing services, which may elicit positive behavioural change at the time of use, reducing some drug-related harms [84].

If pill testing is to be discussed constructively, the final piece of the puzzle is the maintenance of cross-sectoral partnerships. Strong links must be (re)forged between government, police, AOD treatment services and research institutions, as well as with nightclub and music festival industries. There is already movement from within the latter for such partnerships [92, 93]. However, as noted by these groups, the success of any initiative is contingent upon the extent of support from key stakeholders––health, police and government––to serve as ‘drug policy actors’ [11], [5], [94]. These agencies need to lead innovation in thinking and practice, as there remains considerable political capital in the debate that will otherwise impede creation of better drug policy. For example, the police are a critical element in any approach, as to be meaningful, policy must avoid the trap of net-widening and tacitly supporting harm reduction, while allowing police to ‘pick up’ users elsewhere within the system [10, 79]. Harm reduction-oriented policing initiatives must also be clearly defined, well-resourced and widely supported given police play a complex role as an initial contact for many users and conduit for providing case management, links to drug treatment, job training, housing assistance, legal advocacy and counselling [60]. There have been examples of successful initiatives, one of which I will discuss briefly before concluding.

The aforementioned NSEPs and MSIC in Sydney are examples of positive law enforcement-health partnerships. Radical at the time, the trajectory of the relationships between police and healthcare and treatment providers, support services and NGOs provides fertile ground for discussion and the foregrounding of future reforms, as there was a discernible shift in thinking and application that led to positive outcomes for the community (e.g. reduced public drug use and associated ‘litter’) and for users (e.g. safer spaces and access to treatment and support). Indeed, the response to drug use in this particular context shifted from a situation of law enforcement opposition and policing practices that largely undermined the operation of these programs, to one where legislative reforms and organisational policy changes facilitated the effective operation of treatment and support services and their ongoing cooperation with NSW police [51]. For instance, possession of injecting equipment or drug paraphernalia was an offence, creating obvious risks for individuals seeking assistance, as well as the NSEPs or MSIC themselves, as organisations that dispense drug-related equipment and provide information regarding their use, while seeking to create a safer, supervised space for people to use their drugs without police interference. In NSW, the solution was reform of the relevant drug control legislation [95], which permitted health and support service personnel within the NSEPs to provide equipment and information to users, or a supervised space in the MSIC without exposing them to prosecution under the *Drug Misuse and Trafficking Act* (NSW) 1985 [96]. A *Commissioner’s Instruction* was also circulated in NSW in 1988, which shaped police operational practice to follow harm reduction principles, directing police to avoid unnecessary patrols of the areas surrounding the NSEPs and MSIC and to use discretion to prevent the discouragement of users seeking help, while ensuring dealers did not take advantage of the perceived leniency [51].

In summary, what was created was a more supportive, public health-focused environment where users were exempted from prosecution and legal constraints related to drug use and/or possession while on the premises and where discretion was applied in policing the surrounding area. To do otherwise would have undermined the purpose of these important and ongoing policy initiatives, analogues of which have since been implemented in most other jurisdictions. Though there are some clear differences in the rationale and application of these initiatives, the success of NSEPs and the MSIC suggests there is scope for a comparable response to party-drug use, with ongoing collaboration and engagement between law enforcement and health services facilitated through an integrated policy comprising pill testing.

## Conclusion: worth the test?

The problem of drugs—both illicit and illicitly used—is a feature of contemporary social life, for which alternative strategies are needed to reduce the harms for users, their families and the wider community. From analysis of key data and the wider literature, it is evident certain forms of problematic party-drug use are concentrated among a small proportion of young club and music festival attendees, challenging the limits of current Australian drug policy and practice. In these dynamic spaces, party-drugs such as ecstasy and methamphetamines are readily available and widely used, with recent evidence of increased consumption of more potent forms (i.e. MDMA and ice) by young people. Pill testing is needed to monitor the quality/content of drugs used, as well as the rapid rise of NPS, which pose significant risks to users and those who share the social spaces of clubs and music festivals.

Pill testing is not a novel concept; in fact, its objectives are consistent with Australia’s NDS, as well as several extant programs. Notwithstanding a strong philosophical rhetoric of harm minimisation, in practice, government policy remains conservative in its approach, prioritising law enforcement strategies and zero-tolerance policies. This is despite evidence of their limited effectiveness, as well as growing support from experts, academics and the community highlighting the need for an alternative approach. Several national surveys and empirical studies have shown that although drug use is illegal, there is a widespread support that harm reduction and public health-focused strategies are, at least, equally worthwhile. Nevertheless, achievement of these goals requires movement beyond entrenched philosophical and moral arguments, which have historically played a part in producing fragmented and contradictory drug policy. Drawing from Garland [46, 47] and O’Malley [45], it is clear the Australian government is concerned that retreat from a tough stance represents a capitulation in an already failed ‘war on drugs’. This article then shows the need to move away from the politics of drug policy toward more evidence-based strategies to maximise the safety of young people that choose to use drugs who, if given the opportunity to do so more safely, will likely ‘grow out’ of use, without the stigma and harms associated with criminalisation. While unambiguous, zero-tolerance messages are unrealistic and disregard contemporary patterns of youth drug use. In contrast, pill testing offers an alternative message; that drug use *is* dangerous, and informing users about what they are taking and the risks not only demonstrates social responsibility for this marginalised group but also that young people have the capacity for rational decision-making and may desist from drug use because they see the risks for the first time.

Taking a more pragmatic view of harm reduction by expanding measures of effectiveness beyond abstinence, to include increased awareness, reduced consumption and other behavioural changes (e.g. peer information sharing), this article has argued pill testing can be an effective harm reduction tool in a range of contexts, with support for its implementation in Australia and opportunities for its broader application in other countries and drug use settings. Evidence suggests pill testing offers several advantages, facilitating long-term data capture, contributing to knowledge on the nexus between consumption habits and perceptions of use, positively influencing drug markets and overall drug quality, while also enabling essential contact between users and support services. Pill testing also encourages cross-sectoral partnership, greater social inclusion and youth agency (including peer-education and engagement), where the task of harm reduction is understood as a shared social, public health responsibility. Indeed, Australian policy-makers should look to and learn from other policy settings, notably Portugal, with the similarly broad aim of lessening the burdens on healthcare systems, overcrowded criminal justice institutions and families, while also reducing problematic use. In this way, pill testing serves as a platform for more nuanced discussion of drug policy ideas and applications, particularly the need for innovative responses, to avoid the deaths of more young Australians. Australia is in the position to, at the very least, conduct comprehensive trials of pill testing and related strategies (e.g. DIY pill testing kits), to enable evidence-based decision-making. Pill testing cannot eliminate the harms of drug use, but it is not intended to. It represents a model that best functions as one part of a much wider harm reduction strategy, to provide less punitive and more pragmatic responses to drug use for the protection of a generation of young club and music festival attendees, clearly establishing its worth in the Australian drug context.

## Abbreviations

ACIC: Australian Criminal Intelligence Commission
AIHW: Australian Institute of Health and Welfare
AMA: Australia Medical Association
AOD: Alcohol and other drugs
APGDPLR: Australian Parliamentary Group on Drug Policy and Law Reform
DIMS: Drug Information Monitoring System
DIY: Do-it-yourself
EDRS: Ecstasy and Related Drug Reporting System
EMCDDA: European Monitoring Centre for Drugs and Drug Addiction
HIV: Human immunodeficiency virus
IDDI: Illicit Drug Diversion Initiative
MDMA: 3,4-Methylenedioxymethamphetamine
MSIC: Medically Supervised Injecting Centre
NCADA: National Campaign Against Drug Abuse
NDS: National Drug Strategy
NDSHS: National Drug Strategy Household Survey
NGO: Non-Government Organisation
NPS: New psychoactive substances
NSEP: Needle and syringe exchange program

## Footnotes

1 This is likely a response to the increased use of ‘sniffer-dogs’ at recent music festivals, despite considerable criticism and research evidence of their ineffectiveness [27].
